# Supplementary material for: Are carbon emissions trading and green financial instruments synergistic? -Comprehensive quantitative research based on content analysis
Source: PLoS One. 2024 Mar 7;19(3):e0298601. doi: 10.1371/journal.pone.0298601 (PMC10919601; doi:10.1371/journal.pone.0298601)
Supplement: S1 Data — (ZIP) [file pone.0298601.s001.zip › Data/地级市Z_F工作报告.zip!/╔┬╬≈/▒a╝a╩╨/└┤╫╘╣1⁄2╓┌║┼í╛┬φ┐╦╩2╛▌═°í┐_2009.docx]

各位代表：

现在，我代表市人民政府向大会作工作报告，请予审议，并请市政协委员和其他列席人员提出意见。

2008年工作回顾

2008年，是我市发展进程中很不寻常、很不平凡的一年。一年来，在市委的正确领导下，市政府全面贯彻落实科学发展观，积极应对经济环境的复杂变化，全力克服地震灾害带来的各种困难，团结带领全市人民解放思想，开拓创新，抢抓机遇，迎难而上，全面完成了市十三届人大一次、二次会议确定的各项任务，经济社会发展取得了显著成绩。

经济保持快速增长，综合实力大幅提升。

坚持把加快发展作为第一要务，全力支持重点项目建设和大企业大集团、“两区多园”加快发展，全市经济保持了快速增长的良好态势。全年地区生产总值达到714亿元，比上年增长15.5%，创近十年来最高水平。完成财税总收入77亿元，增长15.6%，其中地方财政收入23.9亿元，增长22%。规模以上工业完成增加值289.7亿元，增长22.8%；12户大企业大集团完成工业总产值440亿元，对工业增长的贡献率达到58.3%。高新区实现生产总值180亿元，增长31.6%；蔡家坡开发区和各县区工业园发展势头良好，成为全市经济发展的重要力量。节能减排成效明显，万元生产总值能耗降低4%以上。农村经济稳步发展，新农村建设扎实推进。支农惠农力度进一步加大，全市用于“三农”的支出达到26.2亿元，比上年增加7.4亿元。投资6.5亿元的蒙牛两万头现代化奶牛场项目开工建设，农业产业化龙头企业达到75户。新栽果树20.6万亩，果园总面积超过100万亩。县域经济蓬勃发展，凤翔县跻身陕西经济社会发展十强县。城镇居民人均可支配收入13225元，农民人均纯收入3500元，剔除物价因素，分别增长14.2％和12.6％。第三产业实现增加值205亿元，增长12.4%，被评为最具国际影响力旅游城市和全国金融生态城市。

项目建设实现重大突破，发展后劲显著增强。

坚持把重大产业和基础设施项目作为重点，深入开展项目建设年活动，在抓好年初确定70个重点项目的基础上，又新谋划了19个重大项目，全社会固定资产投资完成451亿元，比上年增长42.6%，保持了投资对经济增长的强劲拉动。全市在建项目中投资10亿元以上的有10个，超过亿元的项目92个。宝烟易地改造、冀东海德堡水泥、东岭电解锌等项目建成投用，大唐热电联产、宝二电二期、宝平高速、西宝高速改扩建等项目开工建设，西宝铁路客运专线、麟北煤田运煤铁路和高速公路专线等项目前期工作基本完成，法门寺合十舍利塔、石鼓山主题公园等项目进展顺利。招商引资成效明显，全年合同引资659.9亿元，到位资金232.1亿元，实际利用外资3349万美元。全年争取中央预算内和国债资金21亿元，比“十一五”前两年的总和还要多。

大城市建设步伐加快，载体服务功能日益完善。

坚持把增强辐射带动能力作为大城市建设的重要内容，积极实施东扩南移北上战略，城市总体规划修编工作进展顺利，率先在全省开展了第二轮土地总体利用规划修编试点。基础设施建设力度进一步加大，蟠龙大桥、新滨河南路建成通车，经一路、宝福路、陈仓大道改造等工程顺利完工，污水处理厂二期、引渭渠市区段综合治理等工程建成投用，陈仓物流园区会展中心、9个县级污水处理厂和垃圾处理场等项目进展顺利。创建节水型城市通过国家考核验收，全国文明城市、国家森林城市创建取得了阶段性成果，荣获全国绿化模范城市称号，摘取“中国人居环境奖”。麟游县荣获国家卫生县城称号，太白县成功创建国家级生态示范县，千阳县、凤县创建活动通过国家预验收。在西北地区首家开通了数字化城管系统，城市精细化、科学化管理走在全省前列，荣获全省信息化工作先进城市。

民生工程进展良好，社会建设全面加强。

坚持把保障和改善民生作为政府工作的基本要求，全面启动了民生八大工程，47个重点项目完成投资32.4亿元，是历年来投资力度最大、群众得到实惠最多的一年。建立了全覆盖、无缝隙的社会保障政策体系，我市农村养老保险模式在全国推广。城乡义务教育阶段学杂费全部免除，4所职教中心、48所农村寄宿制学校顺利完工，宝鸡职业技术学院通过教育部人才培养工作水平评估。城乡医疗卫生服务体系进一步健全，72个社区卫生服务机构全部建成。新福园二期、龙丰苑等廉租住房小区开工建设，保障性住房建设受到省政府表彰。市民中心、妇女儿童中心建成投用，市文化艺术中心、11个县区社保服务中心建设进展顺利。提前两年完成“突破西山”战略五年规划任务，凤县率先实现阶段性脱贫目标，全市11.2万人实现脱贫。农村饮水安全工程加快推进，解决了17万农村居民饮水问题。全市人口自然增长率为4.22‰，眉县、陇县、扶风县创建省级计生优质服务先进县通过验收。以迎奥运为契机，全民健身热潮不断兴起，竞技体育取得新成绩。

抗震救灾取得重大胜利，灾后重建扎实推进。

“5.12”特大地震发生后，我们采取一系列有效措施，迅速调运食品、帐篷、衣被、药品等救灾物资，紧急转移安置群众，对伤员进行免费救治，抢修交通、电力、水利、通讯等基础设施，保证了受灾群众基本生活，保证了中考、高考如期进行，保证了全市社会大局稳定。在抗震救灾取得阶段性成果后，我们及时把工作重点转到恢复重建上，全面落实中央和省委、省政府对灾区的扶持政策，科学编制灾后重建规划，积极争取中、省资金，压缩市级机关行政经费，共筹集各类救灾资金20.4亿元。加强救灾款物监管，确保全部用于受灾群众。帮助3万户灾民新建住房10万多间，基本完成了农村受灾群众住房重建任务，受损市政设施、学校医院、人畜饮水、水库堤防等恢复运行。对“三无”、“三孤”人员发放生活救助金1.75亿元，救灾面粉800多万公斤，受灾群众生活得到妥善安置。

民主法制建设得到加强，依法行政能力明显提高。

自觉接受市人大及其常委会的法律监督、市政协的民主监督，市政府的每一项重大决策，都充分听取和尊重社会各界的意见。建立行政事业性收费、重大公共设施项目规划建设、国企改革方案审批等五项制度，政府行政行为和办事程序进一步公开、透明和规范。公共突发事件应急机制不断完善，社会管理和公共服务职能得到加强。政务公开深入推进，电子政务统一平台建设走在了全省前列。廉政建设和反腐败斗争深入开展，部门和行业风气持续好转，政府机关办事效率明显提高。双拥“六连冠”成果得到巩固，村委会换届工作任务全面完成。安全生产形势总体平稳，信访突出问题得到有效解决。平安宝鸡创建活动深入开展，社会治安公众满意度显著提高，和谐宝鸡建设迈出新步伐。

人事、审计、统计、档案、物价、地志、人防、气象、侨务以及妇女儿童、民族宗教、扶残助残、慈善事业、台湾事务等各项工作都取得了新成绩。

各位代表，过去一年，尽管经济形势错综复杂，但是我们始终把加快发展作为主题，保持了奋发有为的精神状态，紧紧抓住灾后重建、扩大内需的政策机遇，上项目、争投资，解难题、促发展，谋划了一批事关宝鸡长远发展的大项目，解决了长期以来困扰宝鸡发展的项目不足、土地瓶颈制约等问题，全市发展进入全新的阶段；尽管特大地震灾害突如其来，但是我们始终把保护人民群众生命财产安全放在首位，做到了临危不乱，沉着应对，政府部门高效运转，干部群众众志成城，有力有序有效地抗击地震灾害，把灾害损失降到了最低程度，通过扎实推进灾后重建，农村困难家庭住房、农村教育卫生等公共服务设施条件得到较大改善；尽管新一届政府面临诸多困难，但是我们始终把科学发展作为己任，牢记使命，履行职责，内聚合力，外树形象，在继承中创新，在创新中发展，既抓当前又谋长远，既突出重点又全面推进，宝鸡正朝着科学发展、加快发展、和谐发展的目标迈进。

2008年的成绩来之不易，应当倍加珍惜。这些成绩的取得，是市委总揽全局、正确领导的结果，是市人大、市政协鼎力支持、共谋发展的结果，是全市人民团结奋斗、辛勤劳动的结果。在此，我代表市政府，向全市广大工人、农民、干部和知识分子，向给予政府工作大力支持和监督的人大代表、政协委员、各民主党派、工商联、各人民团体、社会各界人士，向人民解放军、武警驻宝部队全体官兵、公安干警，表示崇高的敬意和诚挚的感谢！

在肯定成绩的同时，我们也要清醒地看到，当前国际金融危机还在蔓延、仍未见底，经济增速持续下滑，外部经济环境更加严峻，就业压力加大，社会矛盾突出，财政减收增支因素增多，是进入新世纪以来最困难的一年。与此同时，我市经济社会发展中也存在一些深层次的矛盾和问题，主要是：大企业大集团数量不多，重大项目仍然不足，对经济的支撑作用不强；县域经济特色不明显，整体没有突破，城乡差距仍在扩大，农民持续增收困难；旅游、物流、金融等服务业仍然是经济发展的短腿，影响了宝鸡对外开放和城市活力；支撑大城市发展的城乡基础设施建设滞后，城镇化进程缓慢；社会保障制度在落实层面上还存在不少问题，民生工作需要进一步加强；政府职能转变还不到位，一些政府部门和公务员的大局意识不强，办事效率低下，工作作风飘浮，投资软环境需要进一步改善。为此，我们一定要深刻认识国内外经济形势的严峻性和复杂性，增强危机意识和忧患意识，充分利用有利条件，积极应对各种挑战，努力做好各项工作，绝不辜负全市人民的期望和重托。

2009年政府工作

2009年，是我市抢抓机遇、应对挑战、加快发展的关键一年，我们既要应对金融危机，又要谋求更大发展，工作任务十分繁重。当前，我市发展的挑战与机遇并存，困难与希望同在，经济社会发展的基本面和长期向好的趋势没有改变。从国内形势看，一方面国家把保增长作为宏观调控的基本取向，出台了扩大内需的一系列措施，政策密度之大、覆盖面之广前所未有，项目投资强度之大、资金下拨速度之快前所未有。另一方面“关中—天水经济区”规划进入实施阶段，国家将从产业开发、基础设施建设、生态环境治理等方面给予重点支持。与此同时，东部沿海地区产业升级换代，劳动密集型产业加速向中西部地区转移，这些都为宝鸡加快发展带来了难得机遇。从宝鸡发展实际看，经济结构主要以先进装备制造业为主，外贸依存度较低，发展靠大企业大集团支撑，增长靠投资拉动，相对而言，金融危机对我市的影响有限。特别是经过多年努力，我市已经步入了经济发展的上行通道，积蓄了持续发展的势能，为实现跨越发展奠定了坚实基础。只要我们坚定信心，迎难而上，充分调动各级干部群众的积极性，创造性地开展工作，就没有克服不了的困难，就完全有条件变压力为动力、化挑战为机遇，就一定能够把金融危机带来的不利影响降到最低程度，把国家和省上出台的应对金融危机的政策机遇发挥到最大程度，千方百计推动全市经济社会又好又快发展。

做好今年的政府工作，要以科学发展观为指导，全面贯彻落实党的十七届三中全会、中央经济工作会议和市委十届五次全会精神，始终把加快发展作为第一要务，把项目建设作为第一抓手，把改革创新作为第一动力，把富民惠民作为第一追求，着力在大企业大集团、增加农民收入、大城市建设、园区经济、文化旅游产业五个方面实现新的突破，切实保障和改善民生，推进城乡统筹发展，强化社会管理，保持社会稳定，加快实现“三个率先”步伐，努力建设和谐奋进新宝鸡。在具体工作中，就是要抢抓机遇，强化措施，把保持经济平稳较快增长作为首要任务；就是要科学决策，精心谋划，把项目建设作为战略重点；就是要统筹城乡，关注民生，把提高公共服务水平作为基本要求；就是要开拓创新，提高效率，把群众满意作为工作准则。通过全市上下的共同努力，力争在各地新一轮竞争中抢占先机，缩小差距，提升宝鸡综合竞争力。

2009年，经济社会发展的主要预期目标是：生产总值增长14%；全社会固定资产投资增长30%；地方财政收入同口径增长15%；单位生产总值能耗降低4.3%，二氧化硫排放和化学需氧量分别削减4%和2%；城镇登记失业率控制在4.5%以内；城镇居民人均可支配收入和农民人均纯收入分别增长14%和15%以上；居民消费价格总水平控制在4.5%以内；人口自然增长率控制在5.9‰以内。

着重做好以下八个方面的工作：

（一）积极应对金融危机，保持经济平稳较快增长

用足用好扶持政策保增长。为应对国际金融危机，中央实施了积极的财政政策、适度宽松的货币政策，出台了一系列扩大内需、促进消费、金融支持经济增长的政策措施，制定了汽车、钢铁、装备制造等十大产业振兴规划。省政府在出台支持经济发展政策措施的同时，用于支持经济发展和保障民生的资金大幅增加。我们要紧紧抓住这些难得机遇，及时制定贯彻措施，全面抓好工作落实，在国家文件中找项目，从字里行间抓机遇，争取更多的资金、更多的项目落户宝鸡。

制定落实措施保增长。我们出台了落实关键措施促进农民持续增收的意见，通过加大对“三农”的投入，千方百计增加农民收入；按照“扶大、促小、帮困”的思路，出台了推进大企业大集团发展的意见，提出了“七个优先”、“四个激励”的扶持措施，加速大企业大集团扩张；出台了工业企业应对金融危机的十条意见，着力化解企业面临的困难，帮助企业共渡难关；出台加快工业园区发展的意见，鼓励和引导优势产业向园区集中；出台了加快文化旅游产业发展的意见，创新体制机制，整合资源，加大投入，提高第三产业在经济结构中的比重；出台了支持房地产业发展的意见，全市所有二手房交易费用全免，地下停车场项目免收城市基础设施配套费，100亩以上的地块可分期办理土地使用权证，新开工项目可缓缴规费，放宽住房公积金贷款条件，鼓励干部职工购买多套房产，促进房地产市场健康发展。

加大财政支持保增长。今年，市级财政将安排各类保增长、惠民生的资金12.7亿元，比上年增加4.5亿元。用于支持“三农”专项资金3.56亿元，新增3000万元。用于支持工业经济发展2.65亿元，新增1.63亿元，其中工业企业贷款贴息4000万元，大企业大集团建设1000万元，县区工业园建设5000万元，企业土地使用税列支补助2000万元，中小企业担保基金3000万元。安排专项资金5000万元，支持文化旅游产业发展。向市投资（集团）公司再注入资金7000万元，增加城市基础设施建设投入。用于改善民生的资金达到3.3亿元，比去年新增1.4亿元，其中新增廉租房建设资金9300万元，农村居民养老保险补贴3000万元。同时，今年全市落实增值税转型改革、免征存款利息个人所得税、出口退税、降低住房交易税费等方面的政策，以及取消和停征100项行政事业性收费，可减轻企业和居民负担约13亿元。

强化金融服务保增长。加强对全市金融业发展的统筹协调，鼓励金融机构支持宝鸡经济发展，安排300万元奖励金融机构，力争今年全市贷款净增100亿元以上。积极引进股份制银行，在上海浦发银行进驻营业的基础上，抓紧华夏、交通、民生等银行引进工作。做强市投资（集团）公司和高新区开发总公司，壮大中小企业担保公司，着力解决城市基础设施建设、高新区发展、中小企业融资难的问题。安排300万元专项资金，作为农业保险保费补贴，鼓励专业保险公司发展农业保险，有效化解农民种植、养殖风险。抓好蔡家坡硕丰村镇银行、陇县合作银行试点工作，搞活农村金融市场。选择30户企业，加强与深圳、上海创投公司和基金公司合作，争取红旗民爆、宝成仪表、忠诚机床等企业上市。

扩大消费保增长。把增加居民即期消费作为重点，提振消费信心，提升消费能力。完善最低工资制度，不断提高城乡低收入群体的收入水平，让利益分配适当向弱势群体倾斜。扩大居民投资渠道，提高财产性收入，增加消费预期。以燃油税改革为契机，推动汽车等大宗商品消费。深入开展食品药品安全专项整治，让群众放心消费。结合实施“万村千乡工程”，大力推进“家电下乡”、“农机下乡”、“汽车、摩托车下乡”，加快发展连锁超市、购物中心，促进连锁经营向县城发展、向乡镇延伸，开拓农村消费市场。

（二）积极实施大企业大集团战略，推动工业经济强劲增长

全力打造百亿企业集团，壮大工业经济整体实力。大企业大集团是宝鸡工业经济的支柱。结合国家十大产业振兴规划，年内完成汽车、钛材、石油装备、机床工具、铁路装备等重大产业规划编制，加快完善工业产业体系，倾力打造西部先进装备制造业基地。重点抓好陕汽10万辆微卡、宝钛工业园、石油钢管工业园、宝石机械工业园、宝桥扩建、法士特铸造、西凤酒扩建等重大产业项目，促进企业迅速扩大规模，提高竞争能力，年内确保东岭集团完成总收入200亿元，宝石钢管、宝钛集团建成百亿　企业集团。加快培育一批10亿、20亿、30亿的大型企业和企业集团，使宝光、烽火等行业领军企业，发挥优势，提高效益。

大力发展非公经济，培育壮大中小企业群体。中小企业是推动经济发展、吸纳劳动就业的重要力量。围绕装备制造、有色金属、高新技术、能源化工、农产品加工五大基地建设，引导中小企业搞好协作配套，延伸产业链，衍生孵化一批企业，做大产业集群，增强市场竞争力。积极落实税收优惠、金融扶持等政策，着力解决中小企业发展中面临的困难，支持中小企业快速发展。大力开展“农民创家业、能人创企业”活动，鼓励更多的有识之士办企业、搞流通、做老板、当经纪人。今年安排1亿元，增加小额贷款担保金，力争通过贴息方式带动信贷投入10亿元，鼓励下岗失业人员、高校毕业生、复转军人、返乡农民工等各类人员进行创业，推动非公有制经济快速发展。

加快南北二山资源开发，形成新的经济增长点。麟北煤田、凤县铅锌、太白黄金、北山陶土是我市未来发展的资源优势。加大麟北煤田开发力度，加快郭家河井田、太阳寺井田、宝二电二期、大唐热电等项目建设进度，开工建设150万吨甲醇等项目，把宝鸡建成陕西重要的能源化工基地。积极开发凤县有色金属资源，支持东岭锌业等矿产企业快速发展，大力开发电解铅、铅酸蓄电池等下游产品，建设全国重要的铅锌冶炼基地。扩大太白、凤县黄金开发规模，加快鑫坤5吨黄金冶炼项目建设，使宝鸡成为全省重要的黄金基地。在保护生态环境的前提下，开发利用北山陶土、石灰石资源，支持千阳陶瓷工业园、冀东海德堡水泥、千阳海螺水泥等优势骨干企业做大做强，推动宝鸡建材产业快速发展。鼓励、支持、引导各类社会资本，积极参与南北二山矿产资源勘探、开发和利用，增加我市资源储备总量。

发挥科技支撑引领作用，提高自主创新能力。创新是企业的生命。围绕主导产业体系，引进国内外先进技术装备和管理理念，增强全市骨干企业的市场竞争力。加快建立以企业为主体的科技创新体系，积极落实科技创新工作会商制度，支持宝石钢管、秦川集团等企业研发中心建设，着力抓好石油钻采、油气输送装备等科技创新产业化重大专项。扶持科技型中小企业发展，积极应用专利成果，开展产品创新。支持宝桥等企业争创中国名牌产品，在全市培育一批具有核心竞争力的企业和产品。依托重大科技项目、优势企业，引进国内外高层次人才。继续办好企业家“智慧之光”讲座，加快高素质企业家队伍建设。积极开展循环经济单位试点，加快发展新型环保产业，实现资源的高效利用和循环使用。推进工业化与信息化融合，把宝鸡建成富有创新精神和创造活力的创新型城市。

深化国有企业改革，增强发展活力。改革是企业发展的动力。进一步完善公司法人治理结构，加快建立现代企业制度。完成长岭股份与陕西电子信息集团，宝光、陕开与西电集团，宝啤与青啤的战略重组。积极争取陕棉九厂、北照公司等5户企业政策性破产列入国家计划并启动实施。推进商贸流通企业改制重组，总结试点经验，力争全面推开。采取多种方式解决企业债务问题，年内完成5－8户企业债务打包处置。全面完成市属企业所办中小学移交工作。

（三）加快重大项目建设，强力拉动投资增长

抓项目就是抓发展。今年，全市重点建设项目100个，总投资1308亿元，年度计划投资405亿元。按照“保投产、抓续建、促前期、争开工”的要求，以项目建设为突破口，扩大投资规模，确保全年固定资产投资达到586亿元。

推进重大基础设施项目建设，扩大投资规模。以打造西部交通枢纽为目标，构筑高速公路和铁路“大十字”骨架，逐步实现高速公路县县通，形成市域一小时和市中心区半小时交通圈。加快宝平高速、西宝高速改扩建工程进度，确保宝天高速9月份建成通车。做好西宝铁路客运专线、麟北煤田运煤高速公路专线、宝汉高速、法汤高速扩能改造等项目前期工作，力争年内开工建设。继续抓好干线公路网扩建改造、农村公路和城镇小道路建设。加快宝鸡支线机场前期工作，构建宝鸡立体交通网络。加快引红济石、国电750变电站、城乡电网改扩建等工程建设进度，建成一批重大水利、电力项目。紧盯国家拉动内需的4万亿投资，继续加大项目储备争取力度，使更多的项目挤进国家和省上计划盘子，力争全市重点项目储备规模达到2500亿元以上，为我市长远发展奠定坚实基础。

加大招商引资力度，吸引外来投资。把招商引资作为对外开放和加快发展的重要抓手，实行招商引资工作“一把手”负责制。依托企业、工业园区、资源三大载体，围绕现有大企业配套、南北二山资源开发、一村一品、现代服务业，大力开展产业招商、以商招商和委托招商，进一步加大在“珠三角”和“长三角”招商引资的力度。充分发挥驻京办、上海办、深圳办等驻外机构和条管单位的优势，建立长效稳定的招商机制和渠道，不断提高招商引资的实效和水平。积极搭建招商平台，办好法门寺国际文化旅游节、太白山旅游登山节、全球华人省亲祭祖大会和中国西部商品交易会，确保全年实现合同引资600亿元，到位资金240亿元。

（四）全面加强“三农”工作，发展壮大县域经济

以农民增收为目标，推进农业现代化。

大力发展农产品加工和流通型龙头企业、农民专业合作社、农业科技产业园区三大农业市场主体，提升高效畜牧业、优质果业两大产业基地规模化水平，不断完善农技推广服务体系和农产品销售服务网络，做大做强产业，做精做专品牌。围绕主导产业和特色产业，加快建设“一村一品”，积极推进“一县一业”，高标准建设12个现代农业科技示范园。抓好蒙牛两万头现代化奶牛场、12个千头奶牛场、30个标准化奶牛场建设，支持千阳县标准化奶牛养殖小区示范县建设，促进奶业健康发展。继续抓好秦川牛基地和扶风10万头生猪养殖场、凤县百万头生猪大县等建设，推动畜牧业实现扩张性发展。努力打造渭北塬区百万亩优质苹果带、秦岭北麓40万亩优质猕猴桃带和双百万亩花椒、核桃产业带，推进果业突破性发展。全年新增规模以上农业产业化龙头企业10户，发展农民专业合作社60个。

以改善基础设施为重点，推进新农村建设。

抓住国家加大农村基础设施建设投入的机遇，积极争取政策和资金支持，加快农村道路、水利、电力、通讯、市场、信息等基础设施建设步伐，全面改善农村生产生活条件，推动农业发展、农村进步和农民增收。加快农村饮水、灌区改造、除险加固、渭北水利等工程建设，实施土地整治，开展中低产田改造，提高农业综合生产能力。全年新修、改建农村道路1000公里。新建人饮工程210处，解决15万人饮水安全。继续加大“突破西山”力度，启动“全市10万贫困户增收行动计划”，带动南部秦岭山区、西部关山地区、北部乔山地区三大区域板块扶贫开发，做好农村特困户安居项目和贫困地区村级互助试点工作，解决9万农村贫困人口的温饱和脱贫。推进新农村晋星创建活动，建成100个五星村、300个四星村。积极实施“新网工程”，打造农村新型流通网络。加快村级公共服务中心建设，提高农村基层公共服务水平。

以重点镇建设为突破，推进农村城镇化。

抓住省上建设关中城镇群、实施“百镇推进工程”的机遇，加大9个县城和重点建制镇建设力度，加快凤翔撤县设市步伐。力争年内完成全市所有乡镇总体规划和村庄建设规划，加快城镇道路、污水垃圾处理等基础设施建设，搞好绿化、美化、亮化工作，进一步完善城镇功能。加快户籍制度改革，逐步打破城乡户籍界限，吸引更多农民进城落户。认真落实最严格的耕地保护制度和节约集约用地制度，科学合理使用建设用地。

以改革创新为动力，推进农村经济发展。

坚持城乡统筹，推动城乡规划、产业发展、劳动就业、基础设施建设、公共服务等领域的融合，促进公共资源、生产要素均衡配置和自由流动，支持凤县统筹城乡一体化发展试点。建立土地承包经营权流转制度，支持农民发展适度规模经营，大力发展专业村镇，积极培养职业农民。建立城乡统一的建设用地市场，完善征地补偿机制。鼓励农民土地入股参与非公益性项目经营，今年每个县区开展1—2个试点。健全农业投入保障制度，落实各种农业补贴。加快集体林权制度改革，推动林业产业发展。深化粮食流通体制改革，增加粮食储备，保障粮食安全。

（五）加快大城市建设，全面提升城市化水平

科学规划城市布局。

按照“规划引领城市发展，建设完善城市功能，管理提升城市品质，文化彰显城市魅力”的思路，高标准完成新一轮城市总体规划和土地利用规划修编，启动控制性详细规划及专项规划修编，扩大城市规模，实行功能分区，优化城市空间布局结构。以千渭之会地区为重要节点，加大陈仓大道沿线基础设施开发建设力度，加快代家湾地区与虢镇地区对接，在东扩上实现更大突破。加快蟠龙塬新区路、桥、水、电等基础设施建设，为推动城市北上创造条件。以建设西宝客运专线南客站为契机，高标准规划建设秦岭北麓生态新城区，推进城市南移，加快建设百万人口大城市。加快蔡家坡—五丈原地区建设，尽快形成30万人口规模的城市副中心。

不断完善城市功能。

全面实施城市扩容、道路畅通、山水园林、靓化提升、温馨安居“五大工程”，加快城市基础设施建设。年内完成中水回用扩建、底店输水管道、高新东区供热管网等项目建设。加快天然气利用二期、城市供热及代家湾地区、高新东区二期供水管网改造工程进度，完善城市中心区服务功能，在城市中心区全部实现集中供热、天然气通达。开工建设蟠龙塬上塬道路、石头河供水等重点工程，力争开工建设城市高速环线、宝晁公路、渭河南部台塬道路、和谐大道等工程。优化公交线网布局，继续加快“三场两站”建设，确保南坡村、行政中心枢纽站建成投用。加快市区停车场和市场建设，着力解决经二路等地区停车难、市场少的问题，完善城市服务功能。

努力提升管理水平。

以创建国家森林城市、国家生态示范市为目标，打造西部最佳宜居城市。建立健全常态化、长效化、信息化管理机制，完善数字化城管系统功能，提高城市管理水平。加强城市管理综合执法，强化城市环境综合整治，做好公用设施管理和维护。抓好敦仁堡等26个旧城改造项目，加快推进东星村等10个“城中村”改造。继续加强渭河、小韦河、千河综合治理，实施千湖国家湿地公园生态项目，打造百公里渭河绿色生态长廊。做好全国节水型社会建设试点工作。加大生态环境建设力度，扎实推进绿色宝鸡建设，力争国家级生态示范市通过验收，创建国家森林城市取得成功。继续抓好全国文明城市创建工作，不断提高市民素质。

（六）加快高新区发展，努力壮大园区经济

强力推动高新区跨越发展。

以建设国家一流创新型园区为目标，推进二期产业聚集，加快三期开发建设，全力打造国家新材料高新技术产业基地，使高新区成为全市自主创新前沿区、高新技术产业密集区、生态工业示范区和现代化的新城区。积极培育创新主体，构建创业孵化体系，促进高新区由投资驱动向创新推动转变。调整管理体制，赋予高新区行政管辖权，建立与跨越发展相适应的管理体制和运行机制。通过托管、代管及合作开发等多种途径，加强与东南沿海城市工业园区的合作，承接发达地区产业转移。提升高新区载体服务功能，加快建设高新医院，确保高新大道三期、高新一小、高新国际中学和大型购物超市建成投用。年内高新区生产总值达到 240亿元，增速继续位居全国54个国家级高新区前列。

支持工业园区突破发展。

围绕大工业配套、高新技术引进、农产品加工和资源开发，积极推动优势产业、优势企业和优势资源向园区聚集，把园区建成基础设施最好、体制机制最活、开放程度最高、承载能力最强、最具活力的发展区域。加快“突破蔡家坡”步伐，推进汽车工业园建设，把蔡家坡建成西部汽车工业城。完善各工业园区公司化运作机制，做实做强投资公司，加快园区开发由政府推动向企业带动转变。继续加大财政支持园区发展的力度，制定园区基础设施建设贷款贴息办法，推动园区基础设施高起点、高标准建设。建立园区选商引资和招商引资互动机制，实施错位发展，立足产业定位，明确主攻方向，壮大特色产业。

（七）突破文化旅游产业，带动第三产业发展

做大做强文化旅游产业。

围绕打造国内一流旅游目的地，打响炎帝故里、东方佛都、秦岭中央公园、西部城市天堂“四大品牌”，形成法门寺太白山综合旅游区、秦岭都市旅游区、周公庙五丈原钓鱼台历史古迹旅游区、关山草原风光旅游区、岭南山水旅游区“五大板块”。进一步理顺重点旅游景区的经营和管理体制， 对涉及管理部门较多的景区实行管委会体制，将旅游资源所有权、管理权与经营权三权分离。成立市文化旅游产业管委会和开发投资总公司，推动重点景区开发建设。完善法门寺景区运行机制，力争用两年时间完成新景区、新镇区、新农村同步规划建设，全力打响“千载佛家圣地，万世人文经典”的陕西第二个文化符号。加快建设中华石鼓园、西府文化大观园、千渭之会生态文化产业园，加强大遗址保护。高标准做好炎帝陵景区规划建设，开工建设北首岭遗址公园。抓好西岐民俗园、西部兰花园等乡村生态旅游示范点，做好关山草原、周公庙、五丈原、钓鱼台、龙门洞等景区旅游宣传促销活动，提升全市旅游整体发展水平。

积极培育商贸物流业。

结合大城市规划，制定全市商贸物流业规划和优惠政策，引进高端物流企业，培育市场主体，促进物流、资金流、信息流向宝鸡汇集，构建大进大出的现代化流通体系。加快建设陈仓物流园区，确保会展中心在9月初建成投用。扶持华誉等重点物流企业发展壮大，建设西部集装箱、东岭金属材料、秦川机电设备、陕汽加工等五大配送中心。加快建设国际万象商业广场、嘉隆国际商城等重点商业网点，引入沃尔玛、大润发等国际知名商贸企业，提升全市商贸服务业的档次。把经二路、经一路、中山路、公园路，建成地域特色鲜明、文化气息浓厚的商业街。加快建设一批档次高、容量大、辐射广的大中型消费品和生产资料批发市场，构建区域性商贸物流中心。

支持现代服务业快速发展。

加快宝鸡中介大厦建设进度，引导法律服务、资产评估、工程监理、认证检测等中介机构向大厦集中，实现一厅式办公、一条龙服务。大力发展科技、会展、服务外包、电子商务、投资咨询等新兴服务业，加快引进先进技术和新型业态，不断提升我市现代服务业的层次。积极发展医疗保健、家政服务、养老托幼和食品配送等服务，完善社区便民服务体系。

（八）以改善民生为重点，全面推进社会建设

推进以保障和改善民生为重点的社会建设，是保增长的出发点和落脚点，也是政府工作的重要责任。今年全市民生工程计划投资46亿元，比上年增加13.6亿元。

优先发展教育事业。

加大教育投入，推进素质教育，促进各类教育协调发展。继续实施义务教育提升工程和教育质量振兴计划，扩大优质教育资源。加快城乡中小学布局调整步伐，抓好农村中心小学和初中寄宿制学校建设，全部取消复式班，促进义务教育均衡发展。加强高中标准化建设，力争年内建成4-6所省级标准化高中。支持宝鸡职业技术学院创建全省示范性高职院校，加快12个县区职教中心建设，促进职业教育健康快速发展。支持宝鸡文理学院建设硕士学位授权院校。全面启动市特殊教育学校建设和宝鸡教育学院迁建工作。落实义务教育阶段教师绩效工资制，提高中小学教师待遇。加大城乡贫困家庭学生上学救助力度，保障经济困难家庭、进城务工人员子女平等接受义务教育。

加快推进医疗卫生事业发展。

抓好国家医疗卫生体制改革试点工作，完善基本医疗和公共卫生服务。建立健全突发公共卫生事件应急处置机制，加强疾病防控和食品药品监管，确保群众饮食和用药安全。抓好乡村两级医疗卫生机构建设标准化试点工作，积极推行乡村卫生机构“一体化”管理模式。大力发展中医药事业，确保凤县、凤翔、陇县中医院扩建项目建成投用。继续开展卫生县城、村镇创建活动，年内成功创建1—2个全国卫生县城和一批省市卫生村镇，确保国家卫生城市通过复检。全面落实农村妇女免费住院分娩补助政策，力争金台区、麟游县分别建成国家级和省级计划生育优质服务县区。

繁荣文化事业。

不断加强非物质文化遗产保护，编制完成市、县两级非物质文化遗产名录。加快“两馆一站一室”建设，继续实施文化信息资源共享工程，加快市广播电视中心、广播电视“村村通”、“信息入村”等工程实施步伐。加大文艺创作力度，推出一批能够反映宝鸡历史文化和改革发展成果的文艺精品。深化“文化惠民”演出活动，丰富群众文化生活。加强网络文化建设和管理。完善城乡体育设施，开展全民健身活动，提高竞技体育水平。

千方百计促进就业。

今年安排2400万元，新开发6000个公益性就业岗位，在乡镇、行政村分别安排3-5名保洁、保绿、治安管理等公益性就业人员。安排1000万元，对企业富余职工，不分企业性质和隶属关系，进行免费技能提升和转岗培训。结合国家加大基础设施建设的实际，多层次举办返乡人员专项招聘会，进一步促进城乡就业。举办2009中国•宝鸡技能人才招聘大会，打响“宝鸡技工”品牌。引导高校毕业生转变就业观念，鼓励到农村、到社区就业。加大农业产业化推进力度，通过农业内部消化安置农村劳动力。对返乡农民工进行免费培训，对农村富余劳动力参加农业实用技术培训，每人补贴400元。年内全市实现城镇新增就业4.1万人、下岗失业人员再就业1.6万人，劳务输出62万人。

提高社会保障水平。

全面推行新型农村养老保险，基本实现全市农村养老保险全覆盖。进一步扩大被征地农民养老保险、城镇居民养老保险试点范围，扩大农民工、城镇灵活就业人员参加社会保险覆盖面。完善城镇居民基本医疗保险制度，提升医疗保险保障水平。提高新型农村合作医疗管理补贴标准，降低群众医疗费用负担。对就业困难人员自谋职业、个人依法缴纳的养老、医疗、失业三项社会保险费给予补贴，并逐步提高标准。对企业招用农民工的社保补贴政策延长至2010年。加大社会化养老体系建设，抓好县区中心敬老院、老年公寓建设，年内建成1—2所县区老年大学、50个村级老年活动室。加快新福园、川陕路、千渭星城等廉租住房小区建设，两年内基本解决低收入人群的住房问题。各县区社保服务中心年底前建成投用，实现计算机网络化办公，方便群众办事。 加大城乡困难群众和特定对象救助力度，实现社会救助“多层次、全覆盖、广普惠”的目标。抓好市军休服务活动中心、市儿童福利院、市流浪未成年人救助保护中心等项目建设。鼓励支持慈善事业发展，为残疾人多办实事。加强法律援助工作，维护弱势群体的合法权益。

推动和谐社区建设。

认真开展和谐社区示范城区创建活动，完善城镇社区服务体系，建成10个社区养老服务中心，加快社区卫生服务机构标准化建设。抓好社会保障、低保救助、社区卫生、技能培训、社区养老等十项便民服务，最大限度地满足居民多层次的需求。加强小区物业管理，为居民创造舒适生活环境。

全面完成灾后重建任务。

多渠道筹措建设资金，进一步加快农村灾民住房、学校、医疗卫生机构等项目建设，启动实施第二批灾后重建项目，完成首批学校和62个异地搬迁点的重建任务，把学校建成最安全、家长最放心的地方。对城镇因灾重建住房的居民，全面落实国家每户2.5万元、低保户再增加1万元的补助标准，并结合安居房、廉租房进行安置。加强灾后重建资金管理，规范资金使用发放，确保不出问题。力争在两年内完成原定三年的灾后重建任务，让受灾群众过上更加美好的幸福生活。

全力维护社会安全稳定。

建立社会稳定评估研判机制，推行市、县、乡三级领导“一联三包”工作责任制，重视维护群众合法权益，及时合理解决群众反映的问题，坚决纠正各种损害群众利益的行为。健全社会矛盾纠纷调处化解机制，引导群众以理性合法的方式表达利益诉求。坚持各级领导干部特别是主要领导干部处理群众来信和接待群众来访制度，服务群众，化解矛盾。深入开展“五五”普法活动，增强全民法制观念。加大道路交通、非煤矿山、危险化学品、人员密集区等重点行业和部位的安全监管力度，有效预防和遏制重特大事故发生。加强社会治安综合治理，健全治安防控体系，努力建设平安宝鸡。加快应急体系建设，果断处置突发事件，提高减灾救灾能力。保障妇女儿童合法权益。抓好国防教育、人民防空、民兵预备役建设，巩固全国双拥模范城建设成果。加快少数民族聚居地区经济社会事业发展，发挥宗教界在构建和谐社会中的积极作用。

加强政府自身建设。

深化行政管理体制改革，加强政府自身建设，是贯彻落实科学发展观的必然要求，是转变政府职能、提高为民服务水平的关键环节，是推动科学发展、促进社会和谐的重要保障。面对严峻形势、繁重任务和管理难度带来的各种挑战，各级政府要按照科学发展观的要求，继续解放思想，创新政府管理，提高行政能力，努力建设顺应时代要求、适应科学发展、符合人民意愿的为民、务实、廉洁、高效政府。

深入开展学习实践科学发展观活动。

各级政府及其组成人员要按照市委的总体部署，紧紧围绕“党员干部受教育、科学发展上水平、人民群众得实惠”这一目标，切实加强理论学习，以科学发展观武装头脑，不断增强贯彻落实科学发展观的自觉性和坚定性。要认真总结改革开放30年的经验，找准影响和制约科学发展的突出问题，办一些群众迫切需要办的实事。通过深入开展学习实践科学发展观活动，努力建设一支政治坚定、业务精湛、勇于开拓、廉洁奉公的公务员队伍，提高各级政府工作人员为民服务的水平。

实行科学民主决策。

科学民主决策是各项工作成功的重要前提。各级政府做决策、干工作，要做到既向上级负责、又向群众负责，既向现实负责、又向历史负责。政府重大决策的形成和执行都要加强调查研究，做到察民情、听民意、聚民智，尊重客观规律，既谋事又成事。越是困难时期和关键时刻，越要坚持科学决策，对有规划、有资金的重大公共设施项目，具备条件的加快建设，尚不具备开工条件的项目，继续做好规划、论证等前期工作，绝不能盲目开工、乱铺摊子。深刻理解中省政策，正确把握工作大局，研究发展规律，瞄准发展趋势，充分发挥市决策咨询委员会作用，增强决策的针对性，提高决策的科学性。

全面推进依法行政。

主动接受人大及其常委会监督，自觉接受人民政协的民主监督，广泛听取社会各界的意见和建议，各级政府都要严格按照法定权限和程序行使权力、履行职责。完善和严格执行政府绩效管理制度和行政问责制度。把改革作为转变政府职能的主要手段，调动社会资源，打破行业界限，用市场经济的方法，推动各行各业改革。积极稳妥地推进政府机构改革，提高政府运行效率。深化行政审批改革，减少和规范行政审批和许可事项，推进政府管理机制和服务方式创新，更好地为基层、企业和社会公众服务。加大政务公开力度，增强政府工作的透明度和公信力，保障人民群众的知情权、参与权、表达权、监督权，让人民群众知道政府在想什么、做什么，赢得人民群众的充分理解、广泛支持和积极参与。

提高政府执行力。

提高执行力既是政府工作的本质要求，也是保证政府决策顺利实现的关键。各级政府及其组成人员要以高度的责任心和事业心，集中精力抓重点工作、抓关键环节，特别是面对今年严峻的经济形势，要把提高工作效率作为各级政府应对经济困难、保持社会稳定、加快发展步伐的重要标志，改进工作方式，打破常规办事，简化手续，特事特办，急事先办，做到定了就办、说了就干、雷厉风行，全心全意为经济社会发展服务。严肃政治纪律，强化政府权威，做到令行禁止，确保政令畅通。加大督办考核力度，提高政府工作的执行实效。切实精简会议，减少文件，用更多的时间抓好工作落实。

强化廉政建设。

加强对权力运行特别是对领导干部、人财物管理使用、关键岗位的监督，重点规范各种行政事业性收费减免、重大公共设施项目建设、土地征用出让、国有资产转让等公共资源权力运行，做到行政权力运行到哪里，监督就落实到哪里，财政资金运用到哪里，审计就跟进到哪里。坚决纠正损害人民群众利益的不正之风，查处违法违纪案件，加大惩治腐败力度。深入开展执法监察、廉政监察、效能监察，积极探索从源头上预防和治理腐败的新举措。今年政府投资力度大、新上项目多，要确保监管到位，绝不能搞劳民伤财的“形象工程”和脱离实际的“政绩工程”，绝不允许利用扩大公共投资为单位和个人谋取私利。坚持勤俭办一切事情，反对铺张浪费，压缩购车用车、会议支出、公务接待、出国考察经费，把节约的资金更多的用于改善民生。

各位代表！抚今追昔，我们深感肩负的使命神圣而光荣；展望未来，我们对宝鸡的发展前景充满信心。让我们在中共宝鸡市委的坚强领导下，紧紧依靠全市人民，以只争朝夕、奋力拼搏的精神状态，调动一切积极因素，战胜一切艰难险阻，谱写宝鸡又好又快发展的新篇章，以优异的成绩迎接共和国成立60周年！
